# Psychogenic non-epileptic seizures and personality disorders

**DOI:** 10.1192/j.eurpsy.2021.1169

**Published:** 2021-08-13

**Authors:** B. Ezquerra

**Affiliations:** Psiquiatría, Hospital Universitario Guadalajara, Guadalajara, Spain

**Keywords:** personality disorder, psychogenic seizures

## Abstract

**Introduction:**

Epilepsy and its psychiatric comorbidities have been studied frequently over the course of the last years. However, few studies have aimed to establish the relationship between psychogenic non-epileptic seizures (PNES) and personality disorders.

**Objectives:**

The aim of the current study is to discuss the relationship between different personality disorders and PNES in comparison to patients diagnosed of epilepsy but no PNES.

**Methods:**

A case of a 48 year old female patient who attends an intensive following unit at a psychiatric day hospital is presented. The patient was diagnosed with epilepsy at 25 years old. In the last 10 years she has grown completely dependent on her family, presenting at least one epileptic seizure or PNES during the day. She attends the psychiatric unit after neurologists diagnose highly frequent PNES with interference in her day to day routine. During her follow-up at the psychiatric unit different personality disorders are considered. Furthermore, PubMed, Web of Science and PsycInfo databases were searched, using a pre-established strategy in order to identify recent related studies. Afterwards, studies were selected in a systematized manner.

**Results:**

According to different studies up to 75% of patients with PNES have a comorbid personality disorder. Borderline personality disorder seems to be the most frequently simultaneous axis II diagnosis.

**Conclusions:**

Psychiatric disorders are more frequent in patients with psychogenic non-epileptic seizures than patients with only epileptic seizures

